# Attitudes, Barriers, and Motivators Toward Daily Walking and a Mobile App to Increase Walking Among Women: Web-Based Anonymous Survey

**DOI:** 10.2196/48668

**Published:** 2024-02-06

**Authors:** Catherine Jones, Shikha Chandarana, Amita Vyas, Melissa Napolitano

**Affiliations:** 1 Milken Institute School of Public Health The George Washington University Washington, DC United States

**Keywords:** mHealth, mobile health, mobile app, walking, physical activity, step counts, women’s health, age, wearable activity tracker, chronic disease, mental health, mobile phone, COVID-19

## Abstract

**Background:**

There are disparities in the prevalence of physical activity (PA) with women engaging in less PA than men, a gap which widens during midlife. Walking is a generally accepted form of PA among women and should be encouraged. Motivations, barriers, and attitudes to engaging in walking change with age, but the influencing factors are not well understood nor are the features of mobile apps that facilitate daily walking.

**Objective:**

This study explores the relationship between age and women’s self-reported motivations, barriers, attitudes, and beliefs toward daily walking. It further assesses attitudes toward features of a mobile app designed to sync with a wearable step tracker to increase and maintain levels of daily walking among women.

**Methods:**

A web-based anonymous survey was completed by 400 women, aged 21-75 years. The 31-item survey captured women’s perceived barriers and motivators toward daily walking and attitudes toward mobile apps to support and maintain daily walking. For analysis, responses to the survey were grouped into 2 categories of women: ages 21-49 years and ages 50-75 years. Bivariate analyses were conducted through SPSS (IBM Corp) for each of the survey questions using chi-square for dichotomous variables and 1-tailed *t* tests for scales and continuous variables to identify significant differences between the groups. One-tailed *t* tests were run for scaled variables to identify significant differences between the 10-year age increments.

**Results:**

Significant barriers to daily walking were observed in the 21-49–year group for personal and work responsibilities, motivational and psychosocial factors, and physical and environmental factors. Motivators to walk daily in the 21- 49–year group were significantly higher to reduce stress and anxiety, and motivators to walk daily in the 50-75–year group were significantly higher to help manage or lose weight and to reduce the risk of chronic illness. Women’s walking preferences, beliefs around their walking behaviors, and their perceived importance of the features of a future mobile app for walking designed specifically for women showed significant variation according to age. When asked about the importance of features for a mobile app, women aged 21-49 years indicated a significantly higher number of positive responses for the following features: digital community support, rewards or point system, and seeing a daily or weekly or monthly progress chart.

**Conclusions:**

Our findings indicate that barriers, motivators, and beliefs around daily walking and the importance of preferred features of a mobile app vary according to women’s ages. Messaging and app features should be tailored to different age groups of women. These study results can be viewed as a foundation for future research and development of mobile health interventions to effectively increase daily walking among women of all ages.

## Introduction

Physical activity (PA) has established benefits of preventing and treating many adverse health conditions in women, such as heart disease, type 2 diabetes [1], osteoporosis [2], depression [3], and anxiety [4]. Current guidelines recommend that adults engage in at least 150 minutes of weekly moderate-intensity aerobic PA, such as walking, and suggest that health benefits can be achieved in bouts as short as 10 minutes [5]. However, despite the overwhelmingly positive evidence for performing regular PA, older adults continue to be underactive, with older women in particular being the most inactive segment of the population [6]. This gender difference, which is observed across the life span, widens during midlife (ages 40-60 years) [7,8]. Regular exercise declines for many women just when menopause-related physiological changes increase their risk of weight gain and chronic diseases [7,9-11].

The percentage of all adults in the United States, aged 18 years and older, who met the Centers for Disease Control and Prevention’s Physical Activity Guidelines for Americans for aerobic PA in 2020 was 22.7% [12]. Percentages by gender of all age groups who met both aerobic activity and muscle strengthening guidelines in 2020 show males at 28.3% and females at 20.4%. The prevalence of PA among females declines with age from 28.7% for ages 18-34 years to 22.7% for ages 35-49 years [12]. Prevalence continues to further decline to 17.6% for ages 50-64 years ending with 10.8% for those aged 65 years and older. The decline in males moves from 41.3% (ages 18-34 years) to 15.3% (65 years and older) [12]. Men are more likely than women to meet both PA guidelines across all age groups [12].

Walking is one of the most effective interventions for reducing rates of chronic disease as well as one of the best, low-cost, easily implemented, widely accessible, moderate-intensity forms of PA [10,13-16]. Furthermore, the low risk of injury can allow individuals to remain active in older age [10]. The effectiveness of walking programs relies on initiatives aligning with women’s key barriers and motivators [7]. Previous research on walking levels in midlife women shows a lack of time as the primary barrier [7,10,14]. This combined with professional obligations and family care responsibilities relegates walking to a low daily priority for many women [7,17]. Health issues, poor motivation, and absence of social support are other barriers noted [7]. Women are more likely than men to a cite lack of social support and other social influences (eg, embarrassment due to being overweight) as obstacles to PA engagement [18,19]. Furthermore, the effectiveness of the role of social support for PA may be influenced by cultural norms, which should be considered when creating groups and matching walking partners [18]. Environmental factors, such as poor weather, lack of walking paths, and safety concerns [10], can inhibit daily walking as well [7,19].

Motivators of daily walking for midlife women include the associated health benefits, greater well-being, reduced stress, enjoyment, social support, and accountability to others [20]. Walking for transport was an important facilitator for some. Midlife women are motivated by immediate enjoyment versus long-term benefits [7]. The social aspect of making friends was a primary motivation for participating in health walks [7,21]. A study involving low-income urban mothers identified social connection as their most powerful facilitator alongside “me time” and the opportunity to gain a brief respite from their responsibilities [22].

Encouraging inactive populations, particularly women, to increase walking is an important public health consideration and remains a challenge [23]. The first step to increasing walking is to accurately measure it [24]. The use of technology, including wearable fitness trackers and smartphones, shows a great deal of promise for measuring and encouraging walking among women [25]. Results of a 2015 feasibility study by Arigo [17] showed that a large proportion of midlife women had purchased or intended to purchase a wearable tracking device for personal use after returning the program device used in the study (16/20, 80%). This continued interest in tracking highlights the potential for longer-term behavior change, particularly with novice users, with commercially available wearable technology [18].

Increasing health issues among youths due to a sedentary lifestyle as well as the growing demand for fitness apps for women are key factors propelling the market growth into a multibillion-dollar business [26]. Fitness apps are designed to motivate and persuade behavior change to help their users achieve health and wellness goals. While the industry of fitness apps for women is rapidly evolving, there is a lack of research on the impact of gender-centered design on users’ adoption, usage, perceptions, retention, and outcomes. This study explores the relationship between age and women’s self-reported motivations, barriers, attitudes, and beliefs toward daily walking. It further assesses attitudes toward features of a mobile app designed to sync with a wearable step tracker to increase and maintain levels of daily walking among women.

## Methods

### Research Design and Participant Recruitment

Our study design involved a web-based anonymous survey hosted on SurveyMonkey only in English. The inclusion criteria were to be female as sex assigned at birth, or intersex identifying as female, and to be 21-75 years of age living in the United States. Exclusion criteria were to be male as sex assigned at birth and to be younger than 21 years or older than 75 years of age. Proof of gender and age was not required. Recruitment for the survey was carried out using a URL link or QR code, which was distributed on the following digital platforms: email listservs, Facebook, LinkedIn, and Reddit. The survey remained open for approximately 4 months, from February to June 2022, until 400 participants consented to take it.

### Ethical Considerations

The “Research on Daily Walking Habits Among Women Ages 21 to 75” was approved by The George Washington University Institutional Review Board on February 3, 2021 (NCR224026). All participants were recruited via digital outreach. Privacy and confidentiality protections included anonymous and deidentified data collection. All questions on the survey were broad enough to avoid any chance of identification of individuals. To ensure informed consent, immediately upon opening the survey, as the first question, participants were greeted and given information about the research to decide whether to complete it or not. The paragraph concluded with the question, “Would you like to continue?” Answering yes opened the survey. Participants could skip questions or opt out of the survey at any time. There was no time limit to complete it. Completion rate by the 400 respondents was 100%. Average completion time was 5 minutes. There was no compensation, and no physical risks were associated with the survey.

### Data Collection

The survey was comprised of 31 questions (Multimedia Appendix 1). The first question was focused on consent to participate, and the next 5 questions were designed to capture sociodemographic information including sex assigned at birth, age, current relationship status, race, Hispanic, Latinx, or Spanish origin, and employment or student status. Questions 7 to 13 covered dog ownership, employment status, chronic disease diagnosis, advice from a doctor on walking, and the use of mobile apps and devices to track steps. Question 14 asked participants if their walking rates changed since the start of the COVID-19 pandemic. Question 15 asked approximately how many steps a participant walks each day. Questions 16 to 21 were designed to glean information on possible barriers and motivators for daily walking. Questions 22 to 30 were aimed at capturing data on the acceptability of features of a mobile app designed for women to increase daily walking. The last question, question 31, asked participants if they would be willing to use a walking app designed for women and to wear an activity-tracking device, such as a Fitbit, to increase daily walking.

### Data Analysis

When the survey results were downloaded, a codebook was created and uploaded to SPSS software (version 28.0.0.0-142; IBM Corp). We analyzed our independent variable, ages of women, and two dependent variables: (1) barriers and motivators toward daily walking and (2) attitudes toward features of a mobile app to increase walking. Data analysis included descriptive statistics to describe respondents’ demographics (eg, age, relationship status, employment status, and diagnosis of chronic illness). All data were analyzed with stratification by age. The first type of stratification was based on dividing the population into 2 groups of ages: 21-49 years (21- to 49-year group) and 50-75 years (50- to 75-year group), respectively. The second type of stratification was based on dividing the population into groups with 10-year age increments. Bivariate analyses were conducted through SPSS for each survey question using chi-square for dichotomous variables and 1-tailed *t* tests for scales and continuous variables to identify significant differences between the 2 groups. One-tailed *t* tests were run for scaled variables to identify significant differences divided by decades.

## Results

### Descriptive Statistics of Demographics

Sociodemographic questions captured in the survey and displayed in Table 1 included information on sex assigned at birth, age, relationship status, race and ethnicity, employment or student status, chronic disease status, doctor’s advice regarding walking, and having a dog that you walk daily. Results showed that all 400 respondents self-identified as female for sex at birth. Most women were in their 20s (n=123, 29.6%), followed by 30s (n=101, 25.4%), 50s (n=78, 19.3%), then 40s (n=52, 13%), 60s (n=32, 8%), and 70s (n=14, 3.5%).

**Table 1:** Survey participant characteristics.

Demographic characteristics			Full sample (N=400), n (%)		Respondents aged 21-49 years (n=276, 69%), n (%)		Respondents aged 50-75 years (n=124, 31%), n (%)
**Relationship status**							
	Single	142 (35.5)		123 (44.6)		19 (15.3)	
	Married	196 (49)		104 (37.7)		93 (75.2)	
	Living with partner	46 (11.5)		43 (15.6)		3 (2.4)	
	Once married	16 (4)		6 (2.2)		10 (8.1)	
Walks a dog			111 (27.7)		64 (23.3)		46 (37.4)
**Employment status**							
	Not employed	51 (12.8)		16 (5.8)		35 (28.2)	
	Part-time employment	42 (10.5)		16 (5.8)		26 (21)	
	Full-time employment	185 (46.3)		128 (46.3)		60 (48.4)	
**Student status**							
	Full-time student	80 (20)		79 (28.6)		1 (0.8)	
	Part-time student	42 (10.5)		40 (14.5)		2 (1.6)	
Diagnosed with a chronic illness			93 (23.3)		56 (20.3)		37 (29.8)

In the 21- to 49-year group, most women were single, while in the 50- to 75-year group, most were married. The majority of the 400 women were White (n=297, 74.3%), followed by Black (n=54, 13.5%), and small percentages were Asian, American Indian or Alaska Native, and others. Only 7.5% (n=30) of the women identified as Hispanic, Latinx, or Spanish origin. In both groups, the majority of women were full-time employed. The 50- to 75-year group had significantly more part-time employment and significantly less students. Women who self-reported chronic disease were 20% (55/276) in the 21- to 49-year group and 29% (36/124) in the 50- to 75-year group. More women owned a dog that they walked daily in the 50- to 75-year group. Almost no women were told by their doctor not to walk 2.3% (n=9), and 39.9% (n=159) of all women were advised by their doctor to increase their daily walking.

### Women’s Current Walking Behaviors

Results of current walking behaviors show that approximately the same percentage of women in both groups use a step-tracking device (n=144, 52% in the 21- to 49-year group vs n=57, 45.9% in the 50- to 75-year group) and a mobile app to track steps (n=90, 32.7% and n=39, 31.7%). The number of approximate daily steps self-reported by women showed that the 21- to 49-year group had significantly higher percentages for less than 2000 steps (n=28, 10.1% vs n=4, 3.2%; *P*=.03). Other differences in step counts were not as significant between the 2 groups: 2000 to 3000 steps (n=42, 15.2% vs n=16, 13%), 3001 to 5000 steps (n=72, 26% vs n=29, 23.2%), and 5001 to 8000 steps (n=80, 28.9% vs n=28, 22.7%). The 50- to 75-year group had significantly higher percentages in the 8001 steps or more (n=83, 30% vs n=22, 17.4%; *P*=.001) category.

**Figure 1:**
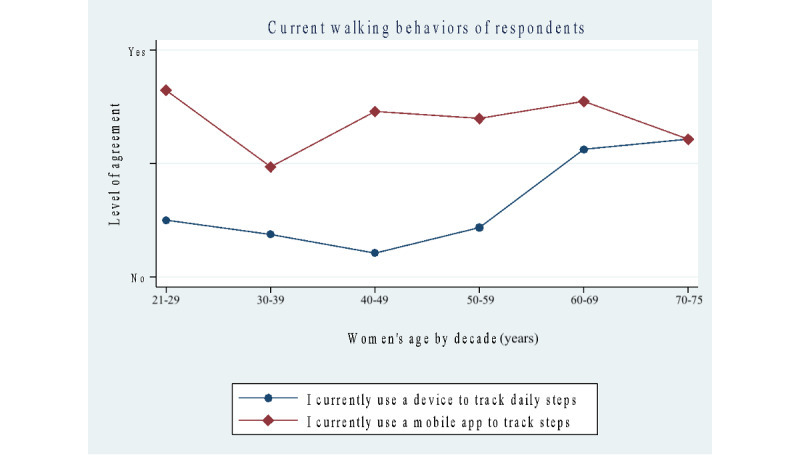
Survey results of step tracking with a wearable device and mobile app for walking.

This survey was conducted from February to June 2022 during the COVID-19 pandemic. Women were asked one question on the survey about COVID-19 to give some context to possible changes in their daily walking habits during the time of the pandemic. Their responses showed that a significantly higher percentage of women in the 21- to 49-year group reportedly reduced their daily walking than the 50- to 75-year group (n=77, 27.9% vs n=15, 12.1%; *P*=.001), while a higher percentage of women in the 50- to 75-year group maintained their amount of daily walking (n=40, 32.2% vs n=55, 19.9%; *P*=.001) or increased it (n=67, 54% vs n=142, 51.4%) during the time period of the survey. Specific groups of women, delineated by age decade, who increased daily step counts during COVID-19 showed that women in their 70s (n=13) self-reported the highest change in walking, and those in their 30s (n=96) self-reported the lowest. Figure 1 illustrates these findings.

### Women’s Barriers to Walk Daily

Regarding barriers to daily walking displayed in Table 2, the 21- to 49-year group had significantly higher percentages than the 50- to 75-year group for the following reasons: personal and work responsibilities (including lack of time, lack of child or older adult care, and work or other scheduling barriers), motivational and psychosocial factors (including lack of motivation to exercise in general, lack of motivation to engage in self-care, lack of support from partner or family, lack of support from work environment, not being able to find a walking group or community to support walking, not enjoying walking or would rather do another form of exercise), and physical or environmental factors (including lack of safe spaces to walk, lack of proper clothing, fear of falling or getting injured, doctor advised not to walk, and difficult or uncomfortable to walk). The most cited barriers with significant differences between the 2 groups, with the 21- to 49-year group higher in all categories, were lack of time (n=172, 62.3% vs n=43, 34.6%; *P*<.001), lack of motivation to exercise (n=119, 43.1% vs n=38, 30.6%; *P*=.02),work or other scheduling (n=115, 41.7% vs n=28, 22.6%; *P*<.001), lack of safe spaces to walk (n=57, 20.7% vs n=7, 5.7%; *P*<.001), and lack of motivation to self-care (n=49, 17.8% vs n=12, 9.7%; *P*=.03). In Table 2, the category “Most cited barriers” illustrates the top 5 barriers to walking by women’s age groups.

**Table 2:** Survey results of women’s barriers to walking daily according to age.

Domains		Full sample (N=400), n (%)	Respondents aged 21-49 years (n=276), n (%)	Respondents aged 50+ years (n=124), n (%)
Personal and work responsibilities^a^		256 (64)	205 (74.3)	51 (41.1)
Motivational and psychological factors^b^		190 (47.5)	145 (52.5)	45 (36.3)
Physical and environmental factors^b^		86 (21.5)	69 (25)	17 (13.7)
None identified^a^		89 (22.3)	43 (15.6)	46 (37.1)
**Most cited barriers**				
	Lack of time^a^	215 (53.8)	172 (62.3)	43 (34.7)
	Lack of motivation to exercise^b^	157 (39.3)	119 (43.1)	38 (30.7)
	Work or scheduling barriers^a^	143 (35.8)	115 (41.7)	28 (22.6)
	Lack of safe spaces to walk^a^	64 (16)	57 (20.7)	7 (5.7)
	Lack of motivation to engage in self-care^b^	61 (15.3)	49 (17.8)	12 (9.7)

^a^*P*<.001.

^b^*P*<.05.

### Motivators for Women to Walk Daily

Motivators to walking daily displayed in Table 3 included the following options: physical factors (feels good physically, gives me more energy, reduces my risk for chronic disease or improves my overall health, burns calories or helps with weight management, and builds strength) and mental or emotional factors (reduces depression, reduces anxiety, reduces stress levels, boosts self-esteem, and allows me to work through issues). Both groups had approximately the same percentage of responses in the categories of feels good physically, gives me more energy, and reduces depression. The 21- to 49-year group had significantly higher percentages than the 50- to 75-year group for the motivator category reduces anxiety (n=181, 65.6% vs n=62, 50%; *P*=.003). While the 50- to 75-year group had significantly higher percentages for walking to burn calories or help with weight management (n=100, 81% vs n=186, 67.4%; *P*=.004) and reduce risk of chronic disease or improve overall health (n=95, 76.6% vs n=168, 61%; *P*=.002). The top 5 motivators to walking by women’s age groups were feels good physically, reduces stress levels, helps with weight management, gives me more energy, and reduces risk for chronic illness or improves overall health as illustrated in the category “Most cited motivators” of Table 3.

**Table 3:** Survey results of motivators for women to walk daily by age groups.

Domains		Full sample (N=400), n (%)	Respondents aged 21-49 years (n=276), n (%)	Respondents aged 50-75 years (n=124), n (%)
Overall physical well-being		387 (96.8)	266 (96.4)	121 (97.6)
Overall mental well-being^a^		344 (86)	244 (88.4)	100 (80.7)
**Most cited motivators**				
	Feels good physically	336 (84)	235 (85.1)	101 (81.5)
	Reduces stress levels	298 (74.5)	213 (77.2)	85 (68.6)
	Helps with weight management^b^	286 (71.5)	186 (67.4)	100 (80.7)
	Gives me more energy	283 (70.8)	194 (70.3)	89 (71.8)
	Reduces risk for chronic illness or improves overall health^b^	263 (65.8)	168 (60.9)	95 (76.6)
	Reduces anxiety^b^	243 (60.8)	181 (65.6)	62 (50)
	Reduces depression	229 (57.3)	164 (59.4)	65 (52.4)

^a^*P*<.05.

^b^*P*<.01.

### Daily Current Walking Routines and Preferences

Results on current and preferred daily walking routines showed that the 21- to 49-year group had higher percentages than the 50- to 75-year group in the categories of taking child or children on walks (n=39, 14.1% vs n=3, 2.4%; *P*<.001), walking for work or use of public transit (n=92, 33.3% vs n=14, 11.3%; *P*<.001), and walking on campus (n=84, 30.4% vs n=7, 5.7%; *P*<.001). The 50- to 75-year group scored highest in the categories of walking the dog (n=48, 39% vs n=69, 25%; *P*=.005) and none of the above, referring to all of the options as motivators to walk (n=59, 48% vs n=35, 28.2%; *P*<.001). Findings in Table 4 show that more women in the 21- to 49-year group prefer to walk alone than in the 50- to 75-year group (n=193, 70% vs n=62, 50%; *P*<.001), while more women in the 50- to 75-year group prefer to walk with a friend (n=66, 53.2% vs n=105, 38%; *P*<.001), or walk with their partner (n=44, 35.1% vs n=76, 27.4%), or with their dog (n=45, 36.2% vs n=72, 26.1%; *P*=.04).

**Table 4:** Survey results of daily current walking routines and preferences by age groups.

Domains		Full sample (N=400), n (%)	Respondents aged 21-49 years (n=276), n (%)	Respondents aged 50-75 years (n=124), n (%)
**Question: Where does walking fit into your daily routine?**				
	Walking the dog^a^	117 (29.3)	69 (25)	48 (38.7)
	Take child or children on walks^b^	42 (10.5)	39 (14.1)	3 (2.4)
	For work or to use public transit^b^	106 (26.5)	92 (33.3)	14 (11.3)
	Part of my job	36 (9)	26 (9.4)	10 (8)
	Walk on campus^b^	91 (22.8)	84 (30.4)	7 (5.7)
	None of the above^b^	137 (34.3)	78 (28.3)	59 (47.6)
**Preferred walking company**				
	Alone^b^	254 (63.5)	267 (69.6)	62 (50)
	With their partner	131 (32.8)	97 (35.1)	34 (27.4)
	With a friend^a^	170 (42.5)	104 (37.7)	66 (53.2)
	In a social group	37 (9.3)	28 (10.1)	9 (7.3)
	With their dog^c^	117 (29.3)	72 (26.1)	45 (36.3)

^a^*P*<.01.

^b^*P*<.001.

^c^*P*<.05.

### Women’s Beliefs Around Increasing Walking Behavior

**Figure 2:**
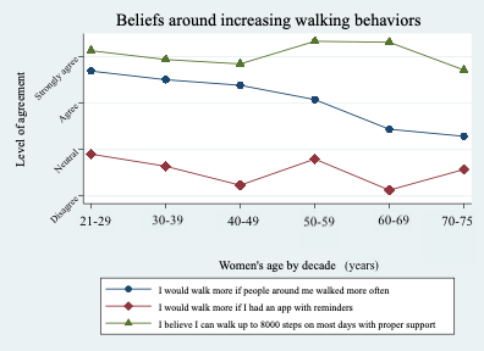
Women’s beliefs around increasing daily walking behaviors according to age decades.

The results of survey questions about beliefs around walking behaviors showed one significant value in the 50- to 75-year group associated with the survey statement: “I would walk more often if people around me walked more often” (scale: 1=strongly disagree to 5=strongly agree). Overall, respondents scored 3.67, while respondents aged 21-49 years scored 3.78, and respondents aged 50-75 years scored 3.41 (*P*<.001). Other survey statements using the same response scale did not show significance: “I would walk more if I had an app with reminders,” all respondents scored 2.83, ages 21-49 years scored 2.84, and ages 50-75 years scored 2.80; and “I believe I can walk up to 8000 steps on most days with proper support,” all respondents scored 4.04, ages 21-49 years scored 4.00, and ages 50-75 years scored 4.12 (Figure 2).

### Women’s Attitudes Toward the Importance of Mobile App Features

Results for survey questions concerning the importance of features for a mobile app showed a significantly higher number of positive responses in the 21- to 49-year group for the following features (scale: 1=low, 2=medium, and 3=high): digital community support (*P*=.03), rewards or point system (*P*<.001), and seeing a daily or weekly or monthly chart of progress (*P*<.001). No significant differences were found for tracking of steps, reminders to walk daily, reminders to wear a step-tracking device, daily in-app motivational messaging, and daily in-app educational messaging.

In Table 5, a deeper analysis of the importance of features on a mobile app was examined by age categories delineated by age decades. The results showed that the youngest age group of women, aged 20-29 years, placed the highest importance on the following features: reminders to walk daily, reminders to wear a step-tracking device, digital community for support (*P*<.05), daily in-app motivational messaging, daily in-app educational messaging, in-app reward or point system (*P*<.001), and daily or weekly or monthly progress reports (*P*<.001). The 50- to 75-year group only placed higher importance on step tracking.

**Figure 3:**
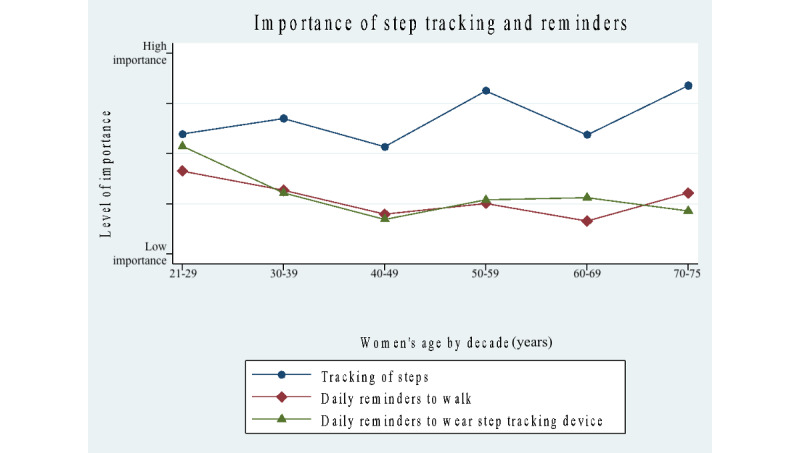
Survey results of the importance of step tracking and reminders for women by age decades.

**Figure 4:**
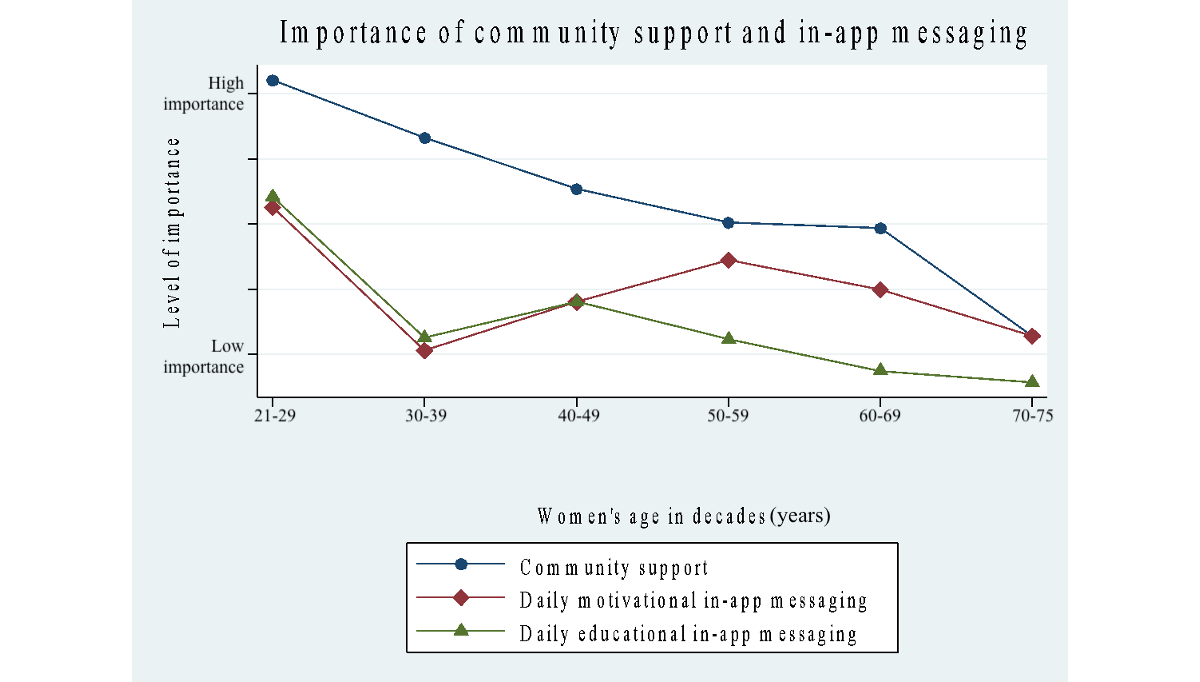
Survey results of the importance of community support and daily messaging by age decades.

**Figure 5:**
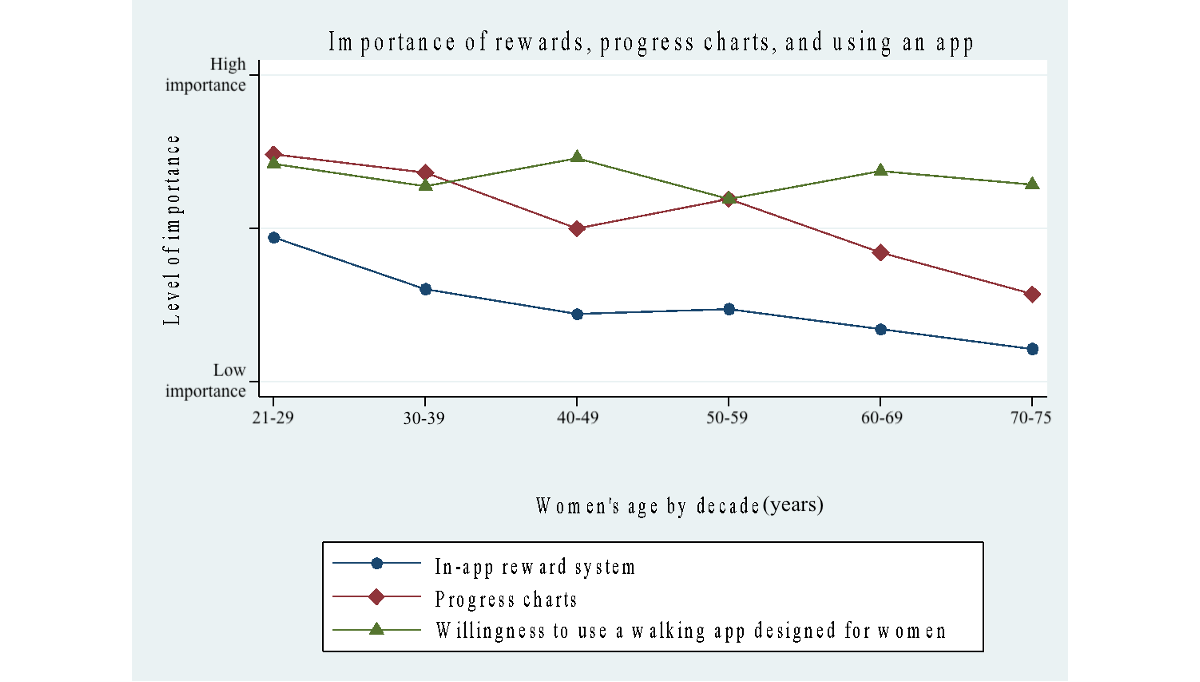
Survey results of the importance of in-app rewards, progress charts, and using an app and step-tracking device for women by age decades.

Figures 3-5 map out mobile app features grouped into different categories: the importance of step tracking and reminders, importance of community support and daily messaging, importance of in-app rewards, progress charts (daily, monthly, and weekly), and willingness to use an app designed for women and to wear a step-tracking device. Our results show that approximately the same percentage of women in both groups use a step-tracking device (n=64, 52% in the younger group and n=127, 46% in the older group), and the same applies to using a mobile app to track daily steps (n=91, 33% and n=40, 32%). Responses to the survey question, “I would be willing to use a walking app designed for women and to wear an activity-tracking device to increase daily walking,” showed overwhelmingly positive responses among all women: yes (n=240, 60%), no (n=55, 13.8%), and not sure (n=105, 26.3%).

**Table 5:** Survey results of the importance of women’s perceptions of mobile app features by age decades.

	Importance levels^a^						
	Full sample	20-29 years	30-39 years	40-49 years	50-59 years	60-69 years	70+ years
Tracking steps	1.9	1.9	1.9	1.8	2.1	1.9	2.1
Reminders to walk daily	1.7	1.7	1.7	1.6	1.6	1.5	1.6
Reminder to wear a step-tracking device	1.7	1.8	1.6	1.5	1.6	1.6	1.6
Build digital community for support^b^	1.7	1.8	1.6	1.5	1.6	1.6	1.6
Daily motivational messaging	1.5	1.6	1.4	1.5	1.5	1.5	1.4
Daily educational messaging	1.5	1.6	1.4	1.5	1.4	1.4	1.4
Reward or point system^c^	1.6	1.9	1.6	1.4	1.5	1.3	1.2
Seeing a daily or weekly or monthly chart of progress^c^	2.3	2.5	2.4	2.0	2.2	1.8	1.6
Willingness to use a walking app designed for women and to wear a step-tracking device	2.4	2.4	2.3	2.5	2.2	2.4	2.3

^a^1=low, 2=medium, and 3=high.

^b^*P*<.05.

^c^*P*<.001.

## Discussion

### Principal Findings

This study explores the relationship between age and women’s self-reported motivations, barriers, attitudes, and beliefs toward daily walking. It further assesses attitudes toward features of a mobile app designed to sync with a wearable step tracker to increase and maintain levels of daily walking among women. Findings indicate that barriers, motivators, and beliefs around daily walking and the importance of preferred features of a mobile app vary according to women’s ages. Therefore, messaging and app features should be tailored to different age groups of women.

### Explanations for Women’s Walking Behaviors

Not surprisingly, women in the 21- to 49-year group, comprised primarily of students, women of childbearing age, young mothers, and women with careers, tended to walk more with children, walk to work or use public transit, or walk on campus. The majority prefer to walk alone, which may be attributed to the need for alone time in their hectic lives, similar to the results of Jones et al’s [21] study in which women wanted “me time” and a respite from their responsibilities. Women in the 50- to 75-year group also preferred to walk alone, but they were more accepting of walking with a friend. One reason might be that more women in the older group have leisure time to walk with friends. In this study, older women were more inclined to walk more if people around them walked more often, which is an important data point to be considered in any walking intervention for older women. These age-focused findings demonstrate the need for more research on gender-tailored fitness apps that satisfy the specific needs of women’s lifestyles at different stages. Catering to women engaged in childcare, for instance, requires communication reminding them to make time for themselves without feeling guilty, while older women might respond well to messaging about walking with a friend to help keep them motivated. There is a dearth of research that systematically examines the acceptability, user experiences, and outcomes of tailored messaging in walking apps for women and the potential impacts they deliver.

### Barriers to Women Walking

The main barriers to walking in the 21- to 49-year group included lack of time, lack of motivation, and work scheduling. This is understandable as many women might be advancing their careers, while also juggling families and other pressures. For the 50- to 75-year group, these same barriers existed but were not as significant and became less significant as age increased. Previous studies have shown lack of time as a primary barrier to PA, as well as professional obligations, family care responsibilities, health issues, poor motivation, and absent social support, which are consistent with our results [7,19]. Addressing these barriers has generated meaningful insights in previous research but has not yet led to a large increase in PA that is sustained over time [25].

This study did not find environmental and safety concerns as common barriers, but these may be related to our sample demographic of mostly White women in either their 20s or 50s. Other studies have shown that in the presence of individual characteristics, such as low self-efficacy and functional limitations, the effects of a prohibitive neighborhood environment on walking behavior in older women were further magnified [6]. More work is desperately needed to address the barriers of environmental concerns, particularly among older women in underserved or unsafe neighborhoods.

### Motivators for Women to Walk

Both age groups of women in this study were motivated to walk because it makes them feel good physically, gives them more energy, and reduces depression, suggesting the powerful physical and mental health benefits of walking. The younger group was more focused on reducing anxiety, while the older group noted walking for burning calories, weight management, reducing the risk of chronic disease, and improving overall health. However, given our concurrent findings of relatively high rates of self-reported chronic disease in the 21- to 49-year group, messaging on chronic disease prevention and management in a walking app should be equally targeted to both groups but in different contexts with tailored nuances.

Menopause increases the risk of weight gain and chronic diseases, happening simultaneously as women’s PA levels tend to decline [27]. Research by Sydora et al [10] found that women experiencing menopause were not averse to regular exercise, especially those seeking to avoid the perceived increased health risks of hormonal therapy, and that walking is the preferred type of exercise among menopausal women. In this study, walking to reduce stress and anxiety was less important for the older group, which supports the results of the Hedgeman et al [28] study on perceived stress across midlife, which found self-reported stress decreased for most women as they transitioned across midlife.

The social aspect of walking, whether to make friends or for social support, has been well received in previous studies, particularly in midlife women [7]. Interestingly, our results showed that social- and family-based walking was not as popular as walking with a friend or walking alone in both age groups of women. A study by Cho et al [29] offers insights into how walking with a partner might motivate walking due to social support; however, it often results in reduced speed that may unintentionally reduce health benefits, a trade-off that needs more research. A feature to build a digital community of support was more favored in the younger group. This is admittedly contradictory, as our data for the 21- to 49-year group showed a preference to walk alone. Furthermore, while the younger group may not want to walk in person with others or they cannot fit it into their schedules, they placed importance on a digital community of support through a mobile app. A study by Hollander et al [20] found that a mobile app for walking (not limited to women) created a digital connection among walking group members, and participants felt that walking improved their mental health, helped to relieve stress, and made them feel more connected with friends or family members. More research should be aimed at determining the effects of sociodemographic variables of women (eg, income, location, age, race, and ethnicity) and their preferences for a digital or in-person community to support walking.

### Acceptance of Technology

The use of smartphones, mobile apps for fitness, and wearable devices to track steps, such as Fitbit, are accepted by women of all ages, and adoption of these devices and exercise apps increases every year [30]. The Pew Research Center concluded that about 1 in 5 Americans use a smartwatch or fitness tracker, with more women than men wearing one (25% vs 18%), and among age groups, more adults aged 18-49 years (25%) wearing one than people aged 50 years and older (17%) [30,31]. In Tong et al’s [32] study, the use of mobile apps and fitness trackers during the COVID-19 pandemic was associated with higher levels of PA in a sample of educated and likely health-conscious individuals among males and females [32]. Our survey responses showed that while an equal percentage of both groups of women wore fitness trackers in general, during COVID-19, higher percentages of women in the younger group reduced their daily walking, as opposed to the older group who maintained or increased steps. More research is needed to ensure walking is maintained among all women during adverse events, such as a pandemic.

Successful interventions to increase women’s daily walking should ideally combine a wearable tracking device with support from mobile app features, such as goal setting, self-monitoring, positive feedback, and social support [9,24,25]. In 2022, findings from a systematic literature review on assessing the acceptability and effectiveness of mobile-based PA interventions for midlife women during menopause concluded that mobile apps and wearable activity trackers showed a small to moderate increase in moderate to vigorous PA among midlife women [27]. The most acceptable features of mobile apps were manual goal setting and step tracking plus the attractiveness and comfort of wearable activity trackers [27].

In this study, younger women placed importance on app features for community support, rewards or a point system, and progress charts, and they are more inclined to use technology for lifestyle interventions, from gaining and redeeming points for coffee and grocery purchases to using apps to track sleep and menstrual cycles. Women in the 21- to 49-year group placed high importance on reminders to walk daily, reminders to wear a step-tracking device, motivational messaging, and educational messaging. The 50- to 75-year group placed the highest importance on step tracking, though they did not place significant importance on progress charts. This may be a technology-based generational difference as more younger women engage with progress charts for academics, digital banking, and other platforms and therefore are more inclined to accept them and to possess the digital literacy to use them with confidence. Daily motivational and educational messaging was more accepted in the younger group perhaps because mini modules of communication, such as text messages and direct messaging on social media platforms, are widely used.

Insufficient exercise among women of all ages is a global public health issue [33]. Walking should be encouraged as much as possible using evidence-based tools to obtain sustainable results. Integrating fitness into health care on a large scale continues to be a challenge, but this is changing as the US health care system is moving more toward a value-based model of care with a focus on prevention and population health versus a fee-for-service model [34]. Today, a prescription for exercise is changing from a doctor simply advising a patient to exercise more to writing out a prescription for exercise, mentioning the type, duration, and frequency [35].

As more doctors are prescribing exercise for chronic health conditions, such as diabetes and hypertension [30], mobile apps connected to wearable devices are playing an increasingly important role in disease management and prevention. Mobile health (mHealth) tools that provide bioinformatic data to doctors and care teams, such as mobile apps and wearable tracking devices, ideally should be supplied or reimbursed by insurers or employee wellness accounts. One example is the United Health Care Rewards plan that provides an app to members, and those who add a fitness tracker, including Apple Watch or other tracking device, have opportunities to earn US $5.25 per week for walking 5000 steps per day and US $8.75 per week for 30 active minutes of fitness a day [36].

Understanding how and why women are motivated to walk will lead to increasingly effective interventions to manage physical and mental health issues at all ages. As the US population aged 65 years and older is projected to nearly double over the next 3 decades, from 48 million to 88 million by 2050 [37], promoting PA among older adults is an important public health, clinical, and economic issue deserving greater attention [27,31,38]. Ideally, future mHealth walking interventions that are uniquely designed for women will combine a wearable tracking device and mobile app with evidence-based behavioral approaches to promote daily walking [39]. This study is among the few focused on women’s walking habits from ages 21 to 75 years old, providing insights into walking behaviors and the motivations and barriers behind them. Our results provide a foundation on which to guide future research and development in this space.

### Strength and Limitations

The major strength of this study was the number of respondents (N=400), all of whom completed the survey 100%. This study had several limitations. First, the survey included self-selected participants who might already be walking enough or would be willing to increase their daily steps. Second, survey question 15 asked approximately how many steps a participant walks each day. Limitations to answering this question include the inherent difficulty to recall and estimate accurately the number of steps one walks daily without using a tracker, which may lead to a lack of reliable data. Inflating daily step counts may also reflect an element of social desirability. Third, the uneven number of participants in each group causes concern as well as the lack of diversity in the sample. Fourth, we did not specifically define chronic disease in the survey. Reporting might be different among women with a chronic disease that significantly reduces mobility, such as rheumatoid arthritis, versus one that does not normally inhibit walking, such as diabetes.

### Conclusions

Insufficient PA is a leading risk factor for noncommunicable diseases and can also negatively affect mental health and quality of life for women of all ages. Findings indicate that barriers, motivators, and beliefs around daily walking and the importance of preferred features of a mobile app vary according to women’s ages. Messaging and app features should be tailored to different age groups of women. These study results can be viewed as a foundation for future research and development of mHealth interventions to effectively increase daily walking among women of all ages.

## Appendix

Multimedia Appendix 1 Web-based survey instrument with 31 questions designed to measure women’s attitudes, barriers, and motivators toward daily walking and the features of a mobile app to increase daily walking.

## Abbreviations

mHealth: mobile health
PA: physical activity

## Data Availability

The data sets generated and analyzed during this study are available from the corresponding author on reasonable request.
