# Supplementary material for: Attitudes, Barriers, and Motivators Toward Daily Walking and a Mobile App to Increase Walking Among Women: Web-Based Anonymous Survey
Source: JMIR Form Res. 2024 Feb 6;8:e48668. doi: 10.2196/48668 (PMC10879972; doi:10.2196/48668)
Supplement: Multimedia Appendix 1 [file formative_v8i1e48668_app1.docx]

| **Question number** | **Question** | **Response categories** |
| --- | --- | --- |
| 1 | Hello! A team of graduate students at the Milken Institute School of Public Health at George Washington University (GWU) are researching rates of daily walking among women. Your participation in this survey will provide us with important information about how much women walk, what motivates them, what barriers they face, and what features of a mobile app would help make walking a daily habit. You are eligible to participate if you are a woman 21 to 75 years old. Your participation in this survey conveys consent. Taking part is completely voluntary and you can quit at any time. This study has been approved by the GWU Office of Human Research (OHR) Institutional Review Board. If you have questions to ask someone from outside the research team, please contact: [ohrirb@gwu.edu](mailto:ohrirb@gwu.edu); 202-994-2715. This is an anonymous survey. We will not collect your name or other identifying information about you. They survey should take about 5 minutes to complete. We feel this survey is reasonable and therefore of minimal risk. If you experience any discomforts, you may opt out of the survey at any time, or you may skip questions. We value your feedback. Please send any questions or comments about the survey to [catherinegjones@gwu.edu](mailto:catherinegjones@gwu.edu). Thanks again! Would you like to continue? | Yes or no |
| 2 | What is your sex assigned at birth? | Male, female, or intersex |
| 3 | I am in my ... check your age. | 20s, 30s, 40s, 50s, 60s, or 70s |
| 4 | How would you describe your current relationship status? | Single, married, cohabitating with partner, or separated, divorced, or widowed |
| 5 | What is your race? Check all that apply. | American Indian or Alaska Native, Asian, Black, African American, Afro Caribbean, or African descent, Native Hawaiian or other Pacific Islander, White, other, prefer not to say |
| 6 | Are you of Hispanic, Latinx, or Spanish origin? | Yes or no |
| 7 | Do you have a dog that you walk daily? | Yes or no |
| 8 | What is your employment status? Check all that apply. | Not employed, employed part-time, employed full-time, full-time student, part-time student |
| 9 | Have you ever been diagnosed with any type of chronic disease? | Yes or no |
| 10 | Have you ever been told by your doctor to increase your walking or exercise level? | Yes or no |
| 11 | Have you ever been told by your doctor to avoid walking? | Yes or no |
| 12 | Do you currently track your steps or physical activity with a device other than your smartphone (smartwatch, Fitbit, or pedometer)? | Yes or no |
| 13 | Do you use a mobile app or virtual coach for walking or exercising? | Yes or no |
| 14 | Since the start of the COVID pandemic, have you been walking ...? | Less, about the same, more, none of the above |
| 15 | Approximately how many steps do you walk each day? | Less than 2,000 steps, 2,000 to 3,000 steps, 3,001 to 5,000 steps, 5,001 to 8,000 steps, 8,001 to 10,000 steps or more, and not sure |
| 16 | There are many reasons people may not walk. Please check any reasons that apply to you. | Lack of time, lack of safe space to walk (sidewalks, lighting), lack of child or elder care, lack of proper clothing, work or other scheduling barriers (work the night shift), lack of motivation to exercise in general, fear of falling or getting injured, lack of motivation to engage in self-care, lack of support from my partner or family, lack of support from my work environment, I can’t find a walking group or community to support me, my doctor has advised me not to walk, it is difficult or uncomfortable to walk, I don’t enjoy walking and would rather do another form of exercise, and none of the above |
| 17 | There are many motivations to walk. Please check any of the reasons that apply to you. | Feels good physically, gives me more energy, reduces my risk for chronic disease and/or improves my overall health, burns calories/helps with weight management, builds strength, reduces depression, reduces anxiety, reduces my stress levels, boosts my self-esteem, allows me to work through issues, and none of the above |
| 18 | Some women walk as part of their daily routines. Check any of the reasons that apply to you. | Walk the dog(s), take my child/children on walks, walk to work or to public transportation to get to work, walking is part of my job, walk to get around on campus, and none of the above |
| 19 | Most days I prefer to walk ... check up to 3. | Alone, with my partner, with a friend, in a group, with my dog(s), and none of the above |
| 20 | I would walk more often if people around me walked more often. | Strongly agree, agree, neither agree nor disagree, disagree, strongly disagree |
| 21 | I would walk more often if I had a mobile app with reminders to walk. | Strongly agree, agree, neither agree nor disagree, disagree, strongly disagree |
| 22 | I believe I can walk up to 8,000 steps on most days with proper support. | Strongly agree, agree, neither agree nor disagree, disagree, strongly disagree |
| 23 | Tracking my steps is of: ... | High importance, medium importance, low importance |
| 24 | Reminders to walk daily is of: ... | High importance, medium importance, low importance |
| 25 | Reminders to wear my tracking device is of: ... | High importance, medium importance, low importance |
| 26 | Community support (virtually connect with other women) is of: ... | High importance, medium importance, low importance |
| 27 | Daily motivational app messaging is of: ... | High importance, medium importance, low importance |
| 28 | Daily educational app tips (benefits of walking) are of ... | High importance, medium importance, low importance |
| 29 | Rewards (point system or confetti) in an app are of: ... | High importance, medium importance, low importance |
| 30 | Seeing a chart of my daily, weekly, monthly progress in an app is of: ... | High importance, medium importance, low importance |
| 31 | I would be willing to use a walking app designed for women and to wear an activity tracking device to increase my daily walking? | Yes, no, not sure |
